# Potent and reversible lentiviral vector restriction in murine induced pluripotent stem cells

**DOI:** 10.1186/s12977-017-0358-1

**Published:** 2017-05-31

**Authors:** Franziska K. Geis, Melanie Galla, Dirk Hoffmann, Johannes Kuehle, Daniela Zychlinski, Tobias Maetzig, Juliane W. Schott, Adrian Schwarzer, Christine Goffinet, Stephen P. Goff, Axel Schambach

**Affiliations:** 1 Institute of Experimental Hematology, Hannover Medical School, Carl-Neuberg-Str. 1, Hannover, Germany; 2 REBIRTH Cluster of Excellence, Hannover Medical School, Hannover, Germany; 3 Institute of Experimental Virology, TWINCORE, Centre for Experimental and Clinical Infections Research, Hannover, Germany; 4 Department of Biochemistry and Molecular Biophysics, Columbia University Medical Center, New York, NY USA; 5 Department of Microbiology and Immunology, Columbia University Medical Center, New York, NY USA; 6 Howard Hughes Medical Institute, Columbia University Medical Center, New York, NY USA; 7 Division of Hematology/Oncology, Children’s Hospital Boston, Harvard Medical School, Boston, MA USA

**Keywords:** HIV-1-based vectors, Murine pluripotent stem cells, Anti-viral restriction, Cyclophilin A

## Abstract

**Background:**

Retroviral vectors are derived from wild-type retroviruses, can be used to study retrovirus-host interactions and are effective tools in gene and cell therapy. However, numerous cell types are resistant or less permissive to retrovirus infection due to the presence of active defense mechanisms, or the absence of important cellular host co-factors. In contrast to multipotent stem cells, pluripotent stem cells (PSC) have potential to differentiate into all three germ layers. Much remains to be elucidated in the field of anti-viral immunity in stem cells, especially in PSC.

**Results:**

In this study, we report that transduction with HIV-1-based, lentiviral vectors (LV) is impaired in murine PSC. Analyses of early retroviral events in induced pluripotent stem cells (iPSC) revealed that the restriction is independent of envelope choice and does not affect reverse transcription, but perturbs nuclear entry and proviral integration. Proteasomal inhibition by MG132 could not circumvent the restriction. However, prevention of cyclophilin A (CypA) binding to the HIV-1 capsid via use of either a CypA inhibitor (cyclosporine A) or CypA-independent capsid mutants improved transduction. In addition, application of higher vector doses also increased transduction. Our data revealed a CypA mediated restriction in iPSC, which was acquired during reprogramming, associated with pluripotency and relieved upon subsequent differentiation.

**Conclusions:**

We showed that murine PSC and iPSC are less susceptible to LV. The block observed in iPSC was CypA-dependent and resulted in reduced nuclear entry of viral DNA and proviral integration. Our study helps to improve transduction of murine pluripotent cells with HIV-1-based vectors and contributes to our understanding of retrovirus-host interactions in PSC.

**Electronic supplementary material:**

The online version of this article (doi:10.1186/s12977-017-0358-1) contains supplementary material, which is available to authorized users.

## Background

In addition to their function as gene transfer vehicles, retroviral vectors can be utilized as tools to investigate specific vector-host interactions. Retroviral vectors are derived from their respective wild-type (wt) viruses by removal of nucleotide sequences in the RNA genomes, which are not essential for transgene delivery. Retroviral vectors are packaged into viral particles composed of structural and enzymatic proteins (Gag-Pol) and follow the natural course of the early retroviral life cycle. This includes cell entry, followed by a poorly-characterized uncoating process of the viral capsid, reverse transcription (RT) of the viral RNA genome into double-stranded DNA, nuclear entry of the viral DNA and stable, proviral integration into the host genome [1].

In this study, we investigated vector-host interactions in pluripotent stem cells (PSC). These cells naturally exist in early embryonic development and give rise to all three germ layers. PSC represent the equivalent to the inner cell mass of the blastocyst and possess major differentiation potential. Among PSC, induced pluripotent stem cells (iPSC) represent a prominent target cell type and cell source as they are easily generated by reprogramming of differentiated cells, a process mediated by ectopic expression of specific transcription factors [2]. After successful reprogramming, iPSC obtain full pluripotency capacity and can differentiate into cells of all three germ layers in vitro as well as in vivo [3]. Therefore, iPSC display a suitable alternative to embryonic stem cells (ESC) for the study of cellular differentiation and disease modeling. Additionally, iPSC are promising resources for cell and gene therapy applications, e.g. innovative cell transplants mediated by retroviral gene transfer.

Retroviral vector application and their gene transfer efficiency are closely dependent on virus-host cell interactions that either support or inhibit efficient infection. Retroviral replication is supported by host co-factors, which are crucial for successful infection. A prominent retroviral host co-factor is cyclophilin A (CypA), which directly binds to an exposed loop of the human immunodeficiency virus type 1 (HIV-1) capsid protein [4, 5]. CypA was described to support early events of HIV-1 infection, such as uncoating and RT initiation [6, 7]. In addition, it was shown that the interaction of CypA with other host co-factors determined the route of nuclear entry and led to alterations in integration site targeting [8]. Furthermore, capsid-associated CypA can shield HIV-1 against cellular recognition and prevent a type 1 interferon response [9]. In contrast, CypA-capsid binding can also inhibit HIV-1 infectivity in a species-specific manner by increasing the sensitivity to certain restriction factors [9–11]. Restriction factors are part of the cell autonomous immunity, are expressed by host cells and inhibit viral replication by inducing viral degradation, transcriptional silencing of integrated proviruses or initiation of anti-viral interferon responses. In this regard, previously identified restriction factors in PSC include the Trim28/ZFP809 complex [12, 13] and YY1 [14]. Both factors target murine leukemia virus (MLV) in murine ESC. However, the role of cell autonomous immunity in PSC is only incompletely understood and so far not described for HIV-1.

Here, we describe an HIV-1-based vector restriction found in iPSC as well as in ESC. Focusing on iPSC, we identified a CypA-dependent restriction mechanism that impaired nuclear entry and reduced proviral integration. The restriction could be decreased by application of high vector doses, by prevention of CypA binding to the HIV-1 capsid or by iPSC differentiation.

## Results

### Murine iPSC exhibit a potent block to HIV-1-based vectors

**Fig. 1:**
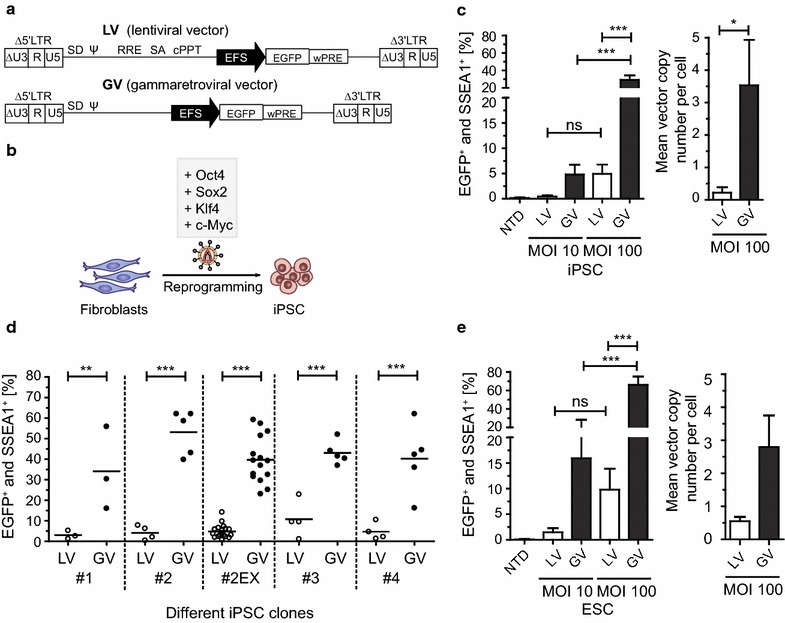
Restriction of HIV-1-based vectors in murine iPSC. **a** Schematic overview of integrated LV and GV used in this study. Vectors contain two LTR with deleted U3 regions (∆U3, self-inactivating design), flanking an EGFP expression cassette driven by an EFS enhancer/promoter. *SD* splice donor, *Ψ* retroviral packaging signal, *RRE* rev responsive element, *SA* splice acceptor, *cPPT* central polypurine tract, *wPRE* woodchuck hepatitis virus posttranscriptional regulatory element. **b** Scheme of reprogramming murine fibroblasts into iPSC by retroviral expression of Oct4, Sox2, Klf4 and c-Myc transcription factors. **c** iPSC transduced with three independently produced viral supernatants of LV and GV at an MOI of 10 and 100 (n = 3). Flow cytometry data of collected EGFP and SSEA1 (pluripotency marker) double positive cells are shown. *NTD* non-transduced control. One-way ANOVA with Tukey-Kramer post hoc test was used for statistical analyses. ns (not significant) p = 0.258; *** p ≤ 0.001 (*left panel*). LV (n = 6) or GV (n = 4) applied to iPSC at an MOI of 100. Mean vector copy numbers per cell were determined 6–8 days after transduction with SYBR Green-based quantitative real-time PCR, based on EGFP copies, and normalized to endogenous PTBP2 DNA copies. The unpaired *t* test with Welch’s correction was used for statistical analysis. * p = 0.017 (*right panel*). **d** LV and GV were applied to different iPSC clones at an MOI of 100. The percentage of EGFP and SSEA1 double positive cells was determined by flow cytometry. One-way ANOVA with Tukey-Kramer post hoc test was used for statistical analyses. #1 ** p = 0.004; #2, #2EX, #3 and #4 *** p ≤ 0.001. **e** LV and GV from three independently produced supernatants were applied to ESC at an MOI of 10 and 100. Analyses were performed 6–8 days after transduction. The percentage of EGFP and SSEA1 double positive cells was measured by flow cytometry. One-way ANOVA with Tukey-Kramer post hoc test was used for statistical analyses. ns p = 0.660; *** p ≤ 0.001 (*left panel*). Mean vector copy numbers per cell were determined with SYBR Green-based quantitative real-time PCR, based on EGFP copies, and normalized to endogenous PTBP2 DNA copies and a plasmid standard (*right panel*)

In this study we analyzed the susceptibility of murine iPSC for vectors derived from HIV-1 (LV; lentiviral vectors) or from Moloney MLV (GV; gammaretroviral vectors). Both retroviral vectors were designed with self-inactivating (SIN) architecture and expressed the enhanced green fluorescence protein (EGFP) reporter gene driven by a short version of the human elongation factor 1 alpha gene (EFS) promoter (Fig. 1a). If not indicated otherwise, we generated vector particles pseudotyped with the vesicular stomatitis virus glycoprotein (VSVg) envelope containing the vector RNA and the retroviral wt replication enzymes and structural proteins. Prior to iPSC transduction, vector supernatants were always titrated on permissive HT1080eCat cells in order to utilize comparable vector doses for following experiments. To compare retroviral transgene transfer into iPSC, LV and GV were applied to iPSC at defined multiplicities of infection (MOI). All iPSC used in this study were generated by ectopic expression of the octamer-binding transcription factor 4 (Oct4), sex determining region Y-box 2 gene (Sox2), Kruppel-like factor 4 (Klf4) and c-Myc in primary fibroblasts (Fig. 1b). A previously characterized and described iPSC clone (clone #2EX) was used throughout this study [15]. In this clone, the reprogramming cassette was excised by Flp recombinase to prevent interference with the integrated lentiviral reprogramming vector during PCR-based analyses. Transduced cells were analyzed for EGFP and anti-stage specific antigen marker 1 (SSEA1) (pluripotency marker in murine cells) co-expression by flow cytometry. Major differences in the susceptibility of murine iPSC for the tested retroviral vectors were observed. Notably, both GV and LV exhibited low transduction rates in iPSC using MOI 10 and 100 (Fig. 1c, left graph). However, LV transduction efficiency remained significantly reduced compared to GV at both MOI. Analyses of the mean vector copy number per cell (detecting EGFP) revealed significantly less integrated vector copies in LV transduced iPSC indicating a pre-integration block (Fig. 1c, right graph). To exclude iPSC clone-specific abnormalities as the basis for the poor permissiveness to LV, we compared clone #2EX (excised reprogramming cassette) to a variety of iPSC clones generated by different reprogramming approaches and derived from various mouse strains. Additionally, we incorporated clones with non-excised reprogramming cassettes, including the parental clone #2 for direct comparison to the #2EX excised version. Remarkably, all tested iPSC clones demonstrated significantly less susceptibility to LV transduction, suggesting that LV restriction is independent from the genetic background and the presence of the reprogramming cassette (Fig. 1d). To analyze whether naturally occurring PSC are susceptible to LV, we tested murine ESC. As similarly observed in iPSC, we measured decreased transduction rates for LV, in contrast to GV, caused by reduced integration events (Fig. 1e).

### LV restriction is observed exclusively in the pluripotent state of a cell

**Fig. 2:**
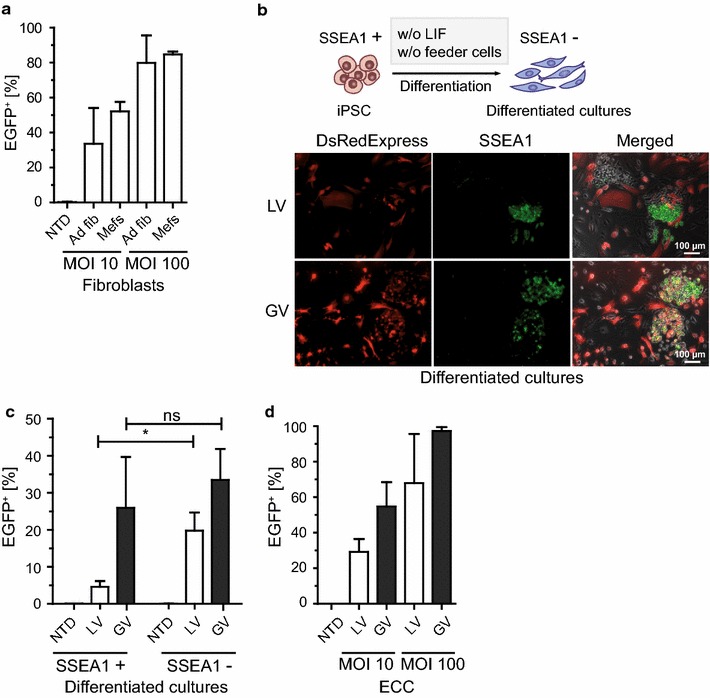
Restriction is observed at the pluripotent cell state. **a** LV were applied to C57BL/6 p14f/f adult fibroblasts (Ad fib) (n = 8) or C3H Mefs (n = 3) at an MOI of 10 and 100 (independently produced viral supernatants). The percentage of EGFP positive cells was determined by flow cytometry. *NTD* non-transduced control. **b** Scheme of iPSC differentiation by withdrawal of LIF and feeder cell co-cultivation (*upper panel*). Representative fluorescence microscopy pictures of transduced differentiated cultures with LV.DsRed or GV.DsRed at an MOI of 100, SSEA1 live staining in *green* and an overlay of all channels are depicted (*lower panel*). **c** Differentiated cultures were transduced with LV (n = 5) and GV (n = 4) encoding EGFP at an MOI of 100 (independently produced viral supernatants). Pluripotent (SSEA1+) and differentiated (SSEA1−) cells were analyzed by flow cytometry. One-way ANOVA with Tukey-Kramer post hoc test was used for statistical analyses. * p = 0.049; ns p = 0.706. **d** ECC transduced with LV (n = 3) or GV (n = 2) at an MOI of 10 and 100 (independently produced viral supernatants). The percentage of EGFP positive cells was analyzed by flow cytometry

Next, we asked, if inhibition of LV gene transfer appears only in iPSC or can also be observed in the parental cells used for reprogramming or in iPSC differentiated progeny. In contrast to iPSC, the parental fibroblasts used for reprogramming, did not show a LV restriction (Fig. 2a). To further test, whether LV restriction is directly correlated with the pluripotent state of the cells, we investigated if the differentiated progeny of iPSC regained permissiveness to LV. We differentiated iPSC by withdrawal of the leukemia inhibitory factor (LIF) and cultured the cells in the absence of supporting feeder cells (Fig. 2b, upper panel). Differentiated cultures were transduced with LV or GV encoding DsRedExpress (LV.DsRed and GV.DsRed). Transduced cells were stained with a green fluorescent SSEA1 antibody and live cell imaging was accomplished by fluorescence microscopy. Almost exclusively SSEA1 negative cells were transduced with LV, whereas SSEA1 and DsRedExpress double positive cells were observed more frequently after transduction with GV (Fig. 2b, lower panel). To better quantify transduction rates, we analyzed differentiated (SSEA1−) or remaining pluripotent (SSEA1+) cells by flow cytometry. In line with the fluorescence microscopy approach, only a small amount of SSEA1+ cells were susceptible to LV in contrast to differentiated SSEA1− cells (4.6 ± 1.6% vs. 19.8 ± 4.9%), while permissiveness of SSEA1+ and SSEA1− cells for GV particles was comparable (26 ± 13.7% vs. 33.5 ± 8.4%) (Fig. 2c). The impact of cell pluripotency on the LV block was further tested using embryonic carcinoma cells (ECC) as an additional pluripotent-like cell model [16]. Interestingly, ECC showed no LV restriction as observed in iPSC or ESC (Fig. 2d).

### LV transduction rate in murine iPSC is enhanced by high vector doses and CSA treatment

**Fig. 3:**
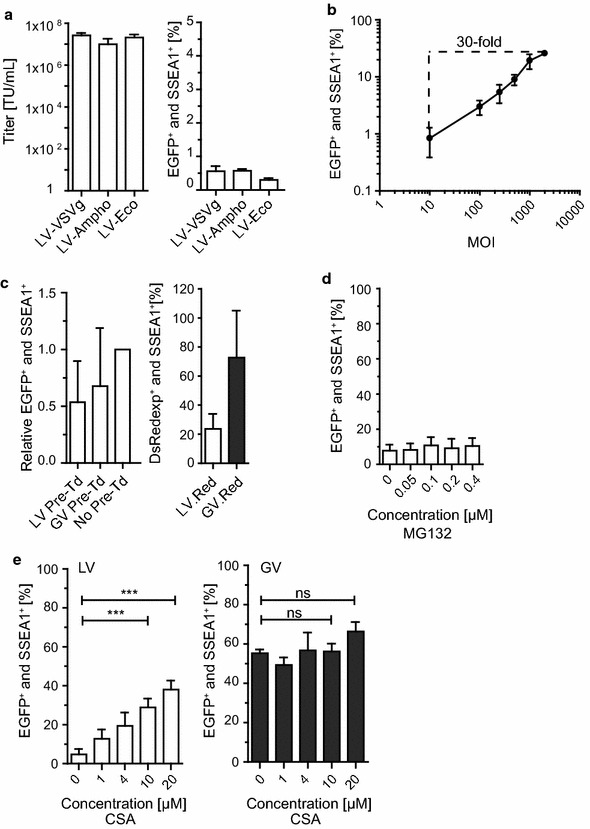
Restriction is circumvented by high MOI or CSA treatment. **a** LV particles pseudotyped with VSVg (LV-VSVg, n = 3), amphotropic (LV-Ampho, n = 3) or ecotropic MLV envelopes (LV-Eco, n = 2) were titrated on permissive HT1080eCat fibroblasts and titers were determined by flow cytometry as transducing units per mL (TU/mL from independently produced viral supernatants) (*left panel*). The different pseudotyped LV particles were applied to iPSC at an MOI of 10 and the percentage of EGFP and SSEA1 double positive cells was assessed by flow cytometry (*right panel*). **b** iPSC were transduced at indicated MOI. EGFP and SSEA1 double positive cells from independent replicates were collected by flow cytometry. MOI 10, n = 5; MOI 100, n = 4; MOI 250, n = 5; MOI 500, n = 5; MOI 1000, n = 5; MOI 2000, n = 4. **c** Pre-transduction (Pre-Td) experiments in iPSC were performed by Pre-Td with LV.Red or GV.Red at an MOI of 1000 (n = 3; independently produced viral supernatants) followed by a second transduction with LV at an MOI of 100, 6 h after Pre-Td. Data of the second transduction are shown relative to no Pre-Td (*left panel*). Transduction of LV.Red and GV.Red are shown as the Pre-Td control (*right panel*). **d** iPSC transduced with LV at an MOI of 100 in the presence of MG132 at indicated concentrations. Data from 3 independently produced viral supernatants were determined by flow cytometry. **e** LV (*left graph*) or GV (*right graph*) were applied to iPSC at an MOI of 100 in the presence of CSA at the indicated concentrations. Data from 3 to 4 independently produced viral supernatants were determined by flow cytometry. One-way ANOVA with Dunnett post hoc test was used for statistical analyses. LV 0 versus 10, *** p ≤ 0.001; LV 0 versus 20, ***p ≤ 0.001; GV 0 versus 10, ns p = 0.999; GV 0 versus 20, ns p = 0.112

It was previously shown that permissiveness of specific cell types to HIV-1 can be enhanced by altering the viral entry route [17, 18]. Therefore, we performed a side-by-side comparison of envelope pseudotypes used for LV particle generation. We tested VSVg-pseudotyped LV (LV-VSVg) and included particles pseudotyped with glycoproteins derived from amphotropic (LV-Ampho) or ecotropic MLV (LV-Eco). While LV-VSVg enters cells via clathrin-mediated endocytosis [19], LV-Ampho employs cell membrane fusion and/or macropinocytosis [20, 21], and LV-Eco enters cells by calveolae-dependent endocytosis and/or direct cell membrane fusion [22–24]. Although LV-VSVg, LV-Ampho and LV-Eco particles obtained comparable titers on permissive HT1080eCat cells (Fig. 3a, left graph), all vectors showed very low transduction rates of iPSC (less than 1%) at an MOI of 10 (Fig. 3a, right graph). Previous work demonstrated the possibility to apply high virus doses to overcome a restriction factor-mediated block [25–28]. Thus, we transduced murine iPSC with LV at increasing MOI. Transduction rates were enhanced from 0.84 ± 0.45% (MOI 10) to 25.83 ± 2.57% (MOI 2000) (Fig. 3b). Despite a 30-fold increase with the highest vector dose, the majority of iPSC remained untransduced, suggesting relatively inefficient abrogation of LV restriction. Next, we performed pre-transduction (Pre-Td) experiments followed by a second transduction to investigate a potential increase in transducability in a second round of transduction. This method is frequently used to provide evidence for a saturable restriction factor, when the factor can be abrogated at a certain MOI of the restricted incoming viral particles [28, 29]. Pre-Td was conducted with DsRedExpress encoding LV.DsRed and GV.DsRed at an MOI of 1000. GV.DsRed Pre-Td served as a control to exclude common cellular defense mechanisms after transduction with high MOI. Following published protocols, a second transduction was then performed with EGFP-encoding LV, at an MOI of 100, 6 h after Pre-Td [25, 28]. Although having conducted successful Pre-Td (Fig. 3c, right graph), Pre-Td resulted in no beneficial effect on LV transduction rates (Fig. 3c, left graph). Next, we tested the small molecules MG132 and cyclosporine A (CSA), which were previously described to enhance lentiviral transduction and counteract lentiviral restriction [11, 30–33]. Dose escalation experiments (using 0.05–0.4 µM) with the proteasome inhibitor MG132 failed to increase LV transduction rates in iPSC (Fig. 3d). Of note, at doses ≥ 0.2 µM, we observed a cytotoxic effect of the drug, resulting in decreased cell numbers as well as in increased AnnexinV and PI positive cells (see Additional file 1). To investigate if abrogation of the CypA-capsid interaction by CSA treatment could circumvent the restriction, as this has been described in other cell types in previous studies [34–37], we transduced iPSC in the presence or absence of different CSA doses. Strikingly, CSA treatment during transduction significantly enhanced LV transduction rates in a dose-dependent manner (Fig. 3e, left graph). In contrast, CSA had no statistically significant effect on GV transduction rates (Fig. 3e, right graph). Of note, while no apoptosis or cell death was observed for any tested CSA concentration, decreased cell numbers were observed with 20 µM CSA (see Additional file 2). Since doses ≤ 10 µM did not affect cell numbers, a concentration of 10 µM CSA was chosen for all further experiments.

### CypA-capsid interaction mediates LV restriction in iPSC

**Fig. 4:**
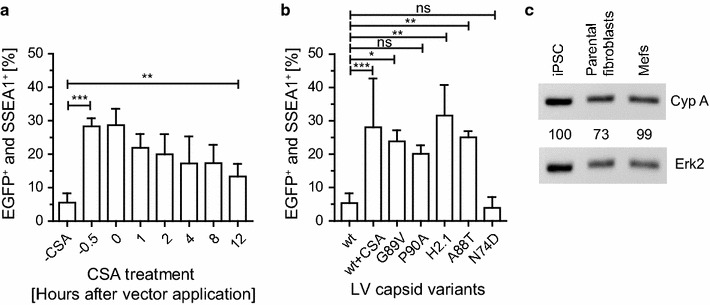
CypA-capsid interaction mediates LV restriction independent from CypA expression. **a** iPSC were transduced with LV at an MOI of 100 in the presence of 10 µM CSA. CSA was added at indicated time points prior to or during transduction. The percentage of EGFP and SSEA1 double positive cells from 3 independently produced viral supernatants were assessed by flow cytometry. Repeated measures one-way ANOVA with Dunnett post hoc test was used for statistical analyses. *** p ≤ 0.001; ** p = 0.003. **b** Transduction of iPSC with LV, harboring wt or mutated HIV-1 capsids. The percentages of EGFP and SSEA1 double positive cells were quantified by flow cytometry (wt n = 9, wt + CSA n = 9; G89V n = 3; P90A n = 3; H2.1 n = 3; A88T n = 5; independently produced viral supernatants). One-way ANOVA with Dunnett post hoc test was used for statistical analyses. wt versus wt + CSA, *** p ≤ 0.001; wt versus G89V, * p = 0.014; wt versus P90A, ns p = 0.066; wt versus H2.1, *** p ≤ 0.001; wt versus A88T, ** p = 0.002; wt versus N74D, ns p = 0.9996. **c** Western blot analysis showing cellular CypA protein levels in iPSC, C57BL/6 p14f/f adult fibroblasts and CF1-Mefs. The percentage of the CypA/Erk2 ratio is depicted for each cell type relative to iPSC

To characterize the dependency of CSA on the time of its addition, we conducted a kinetic study and treated iPSC with CSA at different time points prior to and during transduction. LV transduction without CSA treatment contained equal amounts of DMSO as a solvent control. Interestingly, the strongest CSA effect on LV transduction rate enhancement in iPSC was achieved when adding CSA before (−0.5 h) or at the time of transduction (0 h) (Fig. 4a). Nevertheless, CSA addition still had a significant effect on iPSC susceptibility to LV even when applied 12 h after retroviral vector application. To test whether CypA-capsid interaction mediates the LV restriction or if CSA acts through different mechanisms, we prevented CypA binding to the HIV-1 capsid by using specific CypA-independent HIV-1 capsid mutants. We compared HIV-1 wt capsid and the mutant capsid variants G89V, P90A, H2.1 and A88T for their ability to transduce iPSC. The G89V, P90A and A88T capsid mutants harbor a point mutation causing a single amino-acid change in the CypA binding loop of the HIV-1 capsid, preventing CypA binding to the capsid [36, 38]. Of note, the A88T mutant was shown to prevent binding of CypA and also the myxovirus resistance 2 (Mx2) protein, which was identified as an HIV-1 restriction factor [34–36]. The H2.1 mutant contains a substitution of the equivalent CypA binding loop sequences of HIV-1 to HIV-2, which does not bind CypA [39]. Remarkably, all CypA-independent capsid mutants exhibited clearly improved LV transduction rates (Fig. 4b). In addition, we tested the capsid mutant N74D. N74D harbors a point mutation, which prevents binding of cleavage and polyadenylation specific factor 6 (CPSF6) to the capsid, binds CypA to a lower extent than wt capsids and favors a different nuclear entry route compared to the wt capsid [8, 40, 41]. It was previously reported that the early steps of infection by the N74D capsid mutant virus were inhibited prior to RT in macrophages [42]. Importantly, we did not observe this effect in iPSC, since late RT products using N74D mutant and wt capsid were comparable (see Additional file 3). Interestingly, the N74D capsid mutant did not improve LV transduction (Fig. 4b). To investigate whether the differences in LV transduction efficiencies observed between iPSC and differentiated cell types were due to different intrinsic CypA protein levels, Western blot analyses were used to compare CypA levels in iPSC with their parental fibroblasts and other primary fibroblasts. iPSC displayed comparable CypA levels to the permissive Mefs, indicating that LV restriction was not due to altered CypA protein expression (Fig. 4c).

### Low efficiency of LV transduction in iPSC is associated with reduced levels of 2-LTR circles and proviral DNA integration

**Fig. 5:**
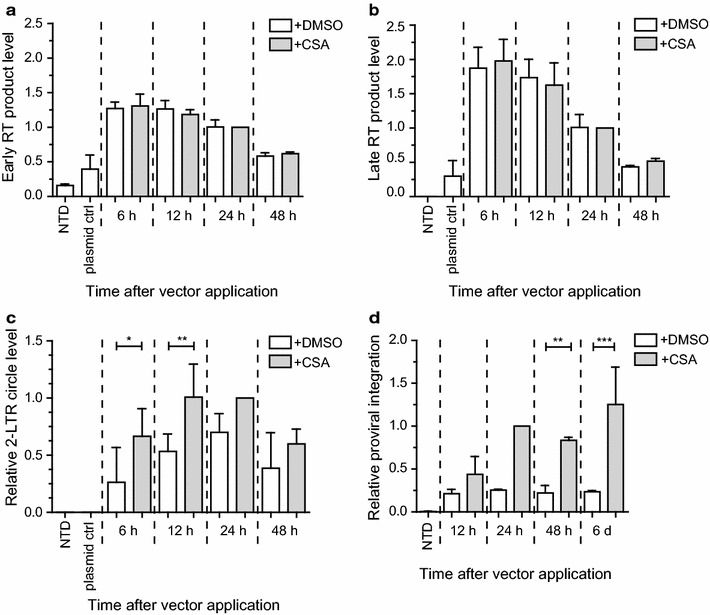
Nuclear entry and integration of LV is reduced in iPSC. iPSC were transduced at an MOI of 10 (with independently produced viral supernatants as indicated) in the presence of 10 µM CSA or an equal volume of DMSO as solvent control. Cells were washed 6 h after vector application and harvested at indicated time points. Data are shown relative to the 24 h time point of LV transduced cells treated with CSA. Samples were analyzed for early RT (n = 3) (**a**), late RT products (n = 3) (**b**), 2-LTR circles (n = 6; * p = 0.030; ** p = 0.005) (**c**) and proviral vector copies (n = 3; ** p = 0.004; *** p ≤ 0.001) (**d**). RT products and 2-LTR circles were evaluated with TaqMan-based quantitative real-time PCR with the $2^{{ - \Delta \Delta Ct}}$ method, normalized to endogenous PTBP2 copies. Proviral vector copies were determined by B1-LTR PCR and obtained values were corrected for plasmid contamination. Repeated measures one-way ANOVA with Tukey-Kramer post hoc test was used for statistical analyses. *NTD* non-transduced control; *plasmid ctrl* plasmid contamination control

We observed strongly reduced vector copies in LV transduced iPSC (Fig. 1c). To characterize the observed LV restriction in greater detail and to identify the step of the early retroviral life cycle affected in iPSC, we analyzed the generation of viral intermediates between cellular entry and integration. iPSC were transduced with LV at an MOI of 10 and emerging RT products were analyzed with TaqMan-based quantitative real-time PCR at different time points. CSA treated cells served as a control for effective LV transduction. RT product levels were normalized to endogenous polypyrimidine tract binding protein 2 (PTBP2) DNA copies. Heat-inactivated supernatants (plasmid ctrl) served as a control for plasmid contamination in the viral vector preparations and DMSO as the solvent control for CSA treated cells. No differences were detected in early RT (Fig. 5a) or late RT product levels (Fig. 5b) when comparing DMSO to CSA treated iPSC. To analyze whether the reverse transcribed viral DNA is imported into the nucleus, we investigated the levels of 2-LTR circles, which are considered as a marker for nuclear entry and are formed in the nucleus by the host cell repair machinery [43–45]. The levels of 2-LTR circles were analyzed by TaqMan-based quantitative real-time PCR (normalized to PTBP2). Primers and probes were designed to detect the LTR-LTR junction region to avoid detection of LV autointegration [45]. In DMSO treated samples, 2-LTR circles were significantly reduced at 6 and 12 h after vector application when compared to CSA-treated cells (Fig. 5c). Next, we included the integrase inhibitor Raltegravir to impede integration and to determine the amount of 2-LTR circles in the presence or absence of CSA. The inhibition of integration by Raltegravir led to significantly higher levels of 2-LTR circles in CSA treated cells, but only a minor increase of 2-LTR circles in DMSO treated cells (see Additional file 4A). Interestingly, treatment of transduced iPSC with Raltegravir and CSA compared to Raltegravir alone led to significantly elevated 2-LTR circle levels. These findings further strengthen the hypothesis that nuclear entry of LV was impaired in iPSC. Strikingly, CSA treatment also significantly increased integrated vector copy numbers (see Additional file 4B). Transduction of Mefs treated under identical conditions served as permissive controls and showed no major effect of CSA on transduction rates in these cells (see Additional files 4A and B). To determine the time point of integration, we analyzed samples from Fig. 5a–c with the B1-LTR PCR, which is the mouse equivalent to the human Alu-LTR PCR. Briefly, proviral integrants were detected with primers detecting the LTR and the B1 repetitive elements in the genome (first stage of PCR), followed by a nested LTR amplification step (second stage PCR). Proviral integration started at 12 h and reached a plateau 24 h after vector application, with no major differences thereafter (as measured at 48 h and a late 6 day time point) (Fig. 5d).

## Discussion

**Fig. 6:**
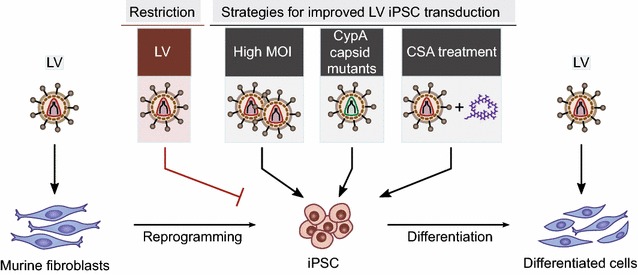
Schematic overview of LV restriction and strategies to improve murine iPSC transduction. Primary fibroblasts of murine origin were efficiently transduced with LV. After reprogramming, iPSC exhibited a potent LV restriction phenotype, which was decreased by high vector amounts, HIV-1 CypA-independent capsid mutants, treatment with CSA during transduction or iPSC differentiation

In this study, we transduced iPSC in order to investigate vector-host interactions in the early retroviral life cycle in pluripotent cell types. Our results revealed that murine iPSC exhibited a potent restriction against HIV-1-based vectors, which was observed after reprogramming fibroblasts into the pluripotent state. Dissecting retroviral intermediates to localize LV restriction revealed functional RT (including early and late RT products), impaired nuclear entry (as measured by 2-LTR circle formation) and reduced proviral integration (as determined by mean vector copy number per cell and proviral integrates). Transduction rates were improved by the use of high vector doses, prevention of CypA binding to HIV-1 capsid (by CSA or using CypA-independent capsid mutants) or differentiation into iPSC progeny (Fig. 6).

The fact that efficiently reverse transcribed proviral DNA of LV failed to integrate into the chromatin of iPSC (Figs. 1c, 5d) suggests inefficient cytoplasmic trafficking, nuclear entry and/or integration. Our 2-LTR circle analyses at different time points after transduction (6, 12, 24, 48 h) revealed significantly reduced levels in untreated compared to CSA-treated cells (Fig. 5c). In addition, cells treated with the CSA/Raltegravir combination exhibited a pronounced increase of 2-LTR circles when compared to cells treated with Raltegravir application alone (see Additional file 4A). These findings clearly support that the mechanism responsible for the LV restriction in iPSC involved inefficient nuclear entry and that CSA relieved this hindrance. This could be explained by unproductive nuclear entry due to misdirected cytoplasmic trafficking to the nucleus, trapping of the PIC or impaired translocation into the nucleus. Alternatively, it is possible that, in addition to the nuclear entry block, there is an intranuclear block (e.g. caused by altered nuclear trafficking or perturbed integration), which is supported by different ratios of 2-LTR circles and proviral integration levels (see Additional file 4A and Fig. 5d). However, we hypothesize that the main block affects the nuclear entry step.

We identified the CypA-capsid interaction as a key event that mediated the restriction. Prevention of CypA-capsid interaction by CSA or specific CypA-independent capsid mutants led to a significant increase in transduction (Figs. 3e, 4b). Our data provide evidence that this interaction is most crucial relatively early after cell entry, but we also observed supportive effects of CSA at the latest time point measured (12 h) (Fig. 4a). Interestingly, CypA expression levels were comparable in restricted iPSC and permissive cells, thus there was no correlation between CypA levels and the block (Fig. 4c). Furthermore, we were unable to saturate the block by pretransduction with high doses (MOI 1000) of LV in our abrogation experiments (Fig. 3c). Potential explanations for the lack of an abrogation phenotype include: (I) even higher concentrations are needed to saturate the potential factor; (II) the block is not saturable; (III) using our doses, other essential cellular pathways and receptors are blocked and are not recycled in a timely manner in the 6 h time window before the second transduction.

The restriction was relieved by use of CypA-independent capsid mutants (P90A, G89V, H2.1, A88T) (Fig. 4b). In contrast, the N74D capsid mutant, which binds CypA to a lower extent than the wt capsid, revealed no beneficial effect on transduction [42]. This suggests that the amount of capsid-bound CypA is important for the restriction. Furthermore, it was previously reported that the capsid mutants P90A and G89V utilize different nuclear entry routes compared to wt capsid [8]. We can therefore envision that, in addition to avoiding binding of CypA molecules to the capsid, also the different nuclear entry routes could play a role in relieving the restrictive phenotype of LV in iPSC.

In addition to a potential restriction factor, the block may also be explained by the lack of a necessary host co-factor. Thus, we performed a detailed microarray analysis of different iPSC clones and their parental fibroblasts and compared expression of previously reported HIV-1 host co-factors for nuclear entry and integration as well as of a set of murine nucleoporins (see Additional file 5). When compared to adult fibroblasts, iPSC exhibited similar or even higher expression levels of examined nucleoporins and important host co-factors for nuclear entry and integration. This suggests that differences in expression levels of the analyzed HIV-1 host co-factors and nucleoporins do not contribute to the underlying mechanism of the observed block.

As previously described, somatic and pluripotent stem cells evolved anti-viral defense mechanisms [12, 14, 31, 46–48]. The novel LV block we identified in iPSC is acquired during the process of reprogramming and relieved upon differentiation (Fig. 2a–c). In our experiments ESC and iPSC exhibited similarly low LV transduction rates and integration events compared to GV (Fig. 1c, e). In contrast, this phenomenon is less pronounced in ECC (Fig. 2d) [16]. This might be explained by the fact that ECC are supposed to represent a more differentiated status compared to PSC, including iPSC and ESC. Thus, we hypothesize that the LV restriction in iPSC may be associated with the pluripotent state.

Although, murine cells are not the natural host for HIV-1, lentiviruses were isolated from several species other than primates and the CypA-capsid interaction was also discovered to be highly conserved [49]. Moreover, the presence of endogenous lentiviruses in the genomes of a variety of different species demonstrates that lentiviruses are more widespread than previously assumed. Therefore, an active lentiviral defense mechanism in murine PSC is imaginable. Likewise, much remains to be discovered regarding anti-viral defense mechanisms in stem cells, especially in PSC. This knowledge can help to develop more efficacious anti-viral treatments in order to modulate cell autonomous immunity, and also increase gene transfer efficiencies for studies in basic biology and therapeutic approaches. Our findings document the characteristics of the restriction against HIV-1 found in PSC and contribute to our understanding of HIV-1-host interactions.

## Conclusions

We identified CypA as a key player in LV restriction in iPSC and discovered impaired nuclear entry and proviral integration as underlying mechanisms for low transduction rates. To the best of our knowledge an HIV-1 block in PSC has not been previously described. Furthermore, the restriction was not observed in parental fibroblasts or differentiated iPSC progeny, which suggests that LV restriction is acquired through reprogramming into the pluripotent state. While our investigation demonstrated a novel HIV-1 restriction in murine PSC, it remained unclear whether the restriction is caused by a restriction factor or the absence of an essential host co-factor. While CypA was previously described to either support or inhibit wt HIV-1 infection in a cell type-dependent manner, the CypA-capsid interaction, we identified to be responsible for the restriction in iPSC, differs from previously described studies and clearly contributes to our understanding of the anti-viral activity in PSC.

## Methods

### Retroviral vector plasmids

Lentiviral vector plasmids pRRL.PPT.EFS.EGFP.pre [50–52] and pRRL.PPT.SFFV.DsRedExpress.pre [53] were referred to as LV and LV.DsRed, respectively. Gammaretroviral vectors pSERS11.EFS.EGFP.pre [52] and pSF91.DsRedExpress [53] were termed GV and GV.DsRed. Except for the LTR-driven GV.DsRed, all vectors had SIN designs with deletions of the viral U3 regions (∆U3), instead containing the internal EFS promoter (short version/250 bp fragment of promoter/enhancer sequences from the human elongation factor 1 alpha gene) or, for LV.DsRed, the SFFV promoter (promoter/enhancer sequences from spleen focus forming virus). For reprogramming, the lentiviral “4-in-1” construct pRRL.PPT.SF.hOKSMco.id.Tom.pre.F+F3, co-expressing codon-optimized versions of the transcription factors Oct4, Klf4, Sox2 and wt c-Myc, was used [15, 54]. The construct contained heterospecific FRT sites (F and F3) to allow Flp recombinase-mediated excision of the reprogramming cassette [15, 55].

### Cells and cultivation

Human 293T (embryonic kidney cell line) [56], human HT1080eCat (fibroblast cell line HT1080 [57] expressing the murine ecotropic receptor mCAT-1 [53]) and the murine ECC line F9 (kindly provided by S. P. Goff, Columbia University Medical Center, New York, NY, USA) were cultured in Dulbecco’s modified Eagle’s medium (DMEM) (Biochrom GmbH, Berlin, Germany) supplemented with 10% heat-inactivated fetal bovine serum (FBS) (GE Healthcare Europe GmbH, Freiburg, Germany), 100 U/mL penicillin, 100 µg/mL streptomycin and 1 mM sodium pyruvate (all from PAN-Biotech, Aidenbach, Germany). CF-1 Mefs (MTI Global Stem, Gaithersburg, MD, USA) and C3H Mefs (kindly provided by T. Cantz, Hannover Medical School, Hannover, Germany) as well as freshly isolated murine adult fibroblasts (C57BL/6 p14f/f) were grown in low glucose DMEM (PAN-Biotech) supplemented with 15% heat-inactivated FBS, 100 U/mL penicillin, 100 µg/mL streptomycin, 2 mM l-glutamine, 1% MEM non-essential amino acids solution (Gibco Life Technologies GmbH, Darmstadt, Germany) and 100 µM beta-mercaptoethanol (Sigma-Aldrich, Munich, Germany) on gelatin pre-coated (0.1% gelatin in phosphate buffered saline (PBS)) wells or flasks. PSC were co-cultured with irradiated (30 Gy) C3H Mefs in ESC medium composed of Knockout DMEM (Gibco Life Technologies) with 15% ESC-tested and heat-inactivated FBS (GE Healthcare Europe GmbH), 2 mM l-glutamine, 100 U/mL penicillin, 100 µg/mL streptomycin, 1% MEM non-essential amino acids, 100 µM beta-mercaptoethanol and 10^3^ U/mL LIF (kindly provided by the Department of Technical Chemistry, Leibniz University Hannover, Hannover, Germany). The following murine PSC were used in this study: iPSC clone #1 (C57BL/6 mouse strain), clones #2, #2EX (excised reprogramming cassette) and #3 (C57BL/6 p14f/f, wt version without conditional knockout of the p14 gene, reprogrammed from adult fibroblasts, mice kindly provided by D. Kotlarz and C. Klein, Ludwig Maximilian University, Munich, Germany), clone #4 (reprogrammed from C3H Mefs) and ESC (C57BL/6 mouse strain, kindly provided by I. Prinz, Hannover Medical School).

### Retroviral particle production

One day before transfection, 5 × 10^6^ 293T cells were seeded per 10-cm dish. Transfection was performed based on the calcium phosphate precipitation method assisted by 25 µM chloroquine (Sigma-Aldrich). For LV or LV.DsRed, packaging cells were co-transfected with 5 μg LV plasmid, 5 μg pRSV-Rev (kindly provided by T. Hope, Northwestern University, Chicago, IL, USA), 1.5 μg pMD.G (VSVg) [58], 12 μg wt pcDNA3.gp.4xCTE (HIV-1 Gag-Pol) [59] or CypA-independent capsid mutant Gag-Pol P90A, G89V, A88T or H2.1 or the N74D mutant [36, 38, 39, 41]. Mutations were introduced by overlap extension PCR and confirmed by sequencing. For production of LV-Ampho(tropic) and LV-Eco(tropic) particles, 2 μg ecotropic [60] or 2 μg amphotropic [61] MLV envelope encoding plasmids were used. To package VSVg pseudotyped GV or GV.DsRed particles, 5 μg GV plasmid, 7 μg pcDNA3.MLV.GP (MLV Gag-Pol) [59] and 1.5 μg pMD.G were co-transfected. Supernatants were harvested 36 and 48 h after transfection, filtered through 0.22 μm pore-size-filters and concentrated (100×) by ultracentrifugation (2 h, 82,740×*g*, 4 °C) (SW32Ti rotor; Beckman Coulter GmbH, Krefeld, Germany). Viral pellets were resuspended in ESC medium and stored in aliquots at −80 °C until further usage.

### Retroviral particle titration and transduction

One day before titration of vector supernatants, 7 × 10^4^ HT1080eCat cells were seeded per well of a 12-well plate. The next day, the culture medium was removed and serial dilutions of vector supernatants were added to the cells. Viral transduction of cells was performed in the presence of 4 µg/mL protamine sulfate and spin-inoculation (1 h, 863×*g*, 37 °C). Three days after transduction, the percentage of fluorescent cells was analyzed by flow cytometry. Vector particle titers were calculated only including samples with < 30% transduced cells to avoid multiple proviral integration events per cell and false titer estimation. Mefs (3 × 10^4^ cells per well of a 12-well plate) or ECC (5 × 10^4^ cells per well of a 12-well plate) were seeded the day before transduction. Specific amounts of vector particles per cell, referred to as MOI, were applied and cells were treated as described above for titration. PSC were transduced in single cell suspension and separated with 0.5% Trypsin-EDTA in PBS (Gibco Life Technologies GmbH) on the day of transduction. Afterwards, PSC were centrifuged (138×*g*, 5 min) and depleted from feeder cells. Depletion was performed by incubating the cells on a 15-cm plate (40 min, 37 °C, 5% CO_2_) to separate the faster attaching feeder from PSC. After applying vector supernatants at specific MOI to 3 × 10^4^ cells (12-well plate) and adding 4 µg/mL protamine sulfate, a spin-inoculation step (1 h, 863×*g*, 37 °C) and an incubation step (1 h, 37 °C, 5% CO_2_) were performed, before PSC were added to feeder cells. The next day, PSC had attached to the feeder cell layer, and the virus-containing medium was replaced by fresh ESC medium. For small molecule supplemented transductions, cells were incubated 30 min prior to and during transduction with indicated concentrations of CSA (Sigma - Aldrich), MG132 (Calbiochem/Merck Millipore, Darmstadt, Germany), Nevirapine (Sigma-Aldrich) or Raltegravir (Santa Cruz Biotechnology, Dallas, TX, USA). Depending on the cell type and experiment, cells were processed by flow cytometry 2 days (differentiated cultures), 3 days (HT1080eCat titration, C3H Mefs), 5–8 days (adult fibroblasts, ESC, ECC, iPSC) or at indicated time points after transduction.

### Reprogramming murine primary fibroblasts to iPSC

Murine fibroblasts from the indicated mouse strains were reprogrammed and characterized as previously described by our group [15, 54].

### Differentiation of iPSC

To induce differentiation, iPSC were depleted from feeder cells, seeded in gelatin pre-coated (0.1% gelatin in PBS) 12-well plates (5 × 10^4^ per well) and cultured in ESC medium without LIF and feeder cells. Medium was exchanged daily and cells were split in the range of 1:5–1:20 on days 3–4 and 5–6. Prior to transduction of differentiated cultures, cells were stained for SSEA1 expression and analyzed by flow cytometry to investigate their stem cell status. Differentiated cultures were transduced with retroviral particles (MOI 100) encoding EGFP or DsRedExpress 6–9 days after starting differentiation. Cells transduced with DsRedExpress encoding vectors were additionally stained with Stain Alive SSEA1 Antibody DyLight 488 according to the manufacturer’s protocol (Stemgent, Cambridge, MA, USA) and analyzed by fluorescence microscopy 2 days after transduction. Pictures were acquired with Axio Observer Z1 (Carl Zeiss AG, Jena, Germany) using Zeiss filter sets 43 (DsRedExpress) and 38 (DyLight 488) and AxioVision 4.8 software. Cells transduced with EGFP encoding vectors were analyzed by flow cytometry 2 days after transduction.

### Flow cytometry

Cells were harvested and subsequently analyzed by flow cytometry (FACS Calibur, Becton-Dickinson, Heidelberg, Germany) using FlowJo software (Tree Star Inc, Ashland, OR, USA). In addition, PSC or differentiated cultures were stained with an Alexa 647 labeled SSEA1 antibody (1.5 ng per sample, 30 min, 4 °C) (eBioscience, San Diego, CA, USA). Samples were pre-gated for viable cells. Data from total viable cell populations with at least 50% SSEA1 positive cells were included in the analyses. The range of SSEA1 positive cells constituted mostly 70–100% throughout the study. The 70–100% range derives from differences between independent transduction experiments, but there were equivalent amounts of SSEA1 positive cells within each experiment. Cells, double positive for SSEA1 and EGFP, are shown in the graphs.

### SYBR Green-based quantitative real-time PCR for detection of mean vector copy number per cell

iPSC were transduced with LV and GV and DNA was isolated 6–8 days after transduction. Vector copies per cell shown in Fig. 1c, e and Additional file 4B were determined by SYBR Green-based quantitative real-time PCR based on EGFP copies (Applied Biosystems, Darmstadt, Germany) using the QuantiTect SYBR-Green PCR Kit (Qiagen, Hilden, Germany) normalized to endogenous PTBP2 (EGFP for: 5′CTATATCATGGCCGACAAGCAGA3′, rev: 5′GGACTGGGTGCTCAGGTAGTGG3′; PTBP2 for: 5′GTCTCCATTCCCTATGTTCATGC3′, rev: 5′GTTCCCGCAGAATGGTGAGGTG3′). Master Mix preparation and PCR were performed as instructed by the manufacturer. Quantification of the mean vector copy number per cell were determined based on the comparative 2^−∆∆Ct^ method and based on a reference plasmid containing EGFP and PTBP2 sequences [62–64].

### Analysis of RT products, 2-LTR circles and proviral integration by TaqMan-based quantitative real-time PCR

Vector supernatants were applied (MOI 10) to iPSC and CF-1 Mefs and remaining vector particles were removed by washing the cells twice with PBS 6 h after transduction. Cells were harvested at indicated time points. To test for plasmid contamination of vector supernatants, equivalent volumes of vector supernatants were heat-inactivated at 65 °C for 1 h and included in the analyses (plasmid ctrl). DNA isolation was performed with the QIAamp DNA Blood Mini Kit (Qiagen) according to the manufacturer’s instructions. For TaqMan-based quantitative real-time PCR (Applied Biosystems), RT products were analyzed for early RT products (strong-stop DNA, for: 5′GCCTCAATAAAGCTTGCCTTGA3′, rev: 5′TGACTAAAAGGGTCTGAGGGATCT3′, probe: 5′AGAGTCACACAACAGACGGGCACACACTA3′), late RT products (U5/downstream PBS, for: 5′TAGTGTGTGCCCGTCTGTTG3′, rev: 5′GAGTCCTGCGTCGAGAGAG3′, probe: 5′TCCCTCAGACCCTTTTAGTCA3′) and 2-LTR circles (junction, for: 5′TAGTGTGTGCCCGTCTGTTG3′, rev: 5′CAGAGAGACCCAGTACAAGC3′, probe: 5′CTCTAGCAGTAACTGGAAGGGCT3′). PTBP2 served as a housekeeping control (for: 5′TCTCCATTCCCTATGTTCATGC3′, rev: 5′GTTCCCGCAGAATGGTGAGGTG3′, probe: 5′ATGTTCCTCGGACCAACTTG3′). RT products were relatively quantified using the comparative 2^−∆∆Ct^ method [62, 63]. PCR was performed at 50 °C for 2 min and at 95 °C for 20 s followed by 60 cycles of 5 s at 95 °C, 20 s at 56 °C and 20 s at 65 °C.

### B1-LTR PCR for analysis of proviral integration

Briefly, a first stage PCR was run with a LTR forward primer, which was designed to harbor a lambda-phage heel sequence at the 5′ end and LTR sequences at the 3′ end. In addition, two outward facing primers binding the highly redundant consensus sequence within the mouse B1 repetitive element were used in the first stage PCR. This was followed by a second stage TaqMan-based quantitative real-time PCR, which amplified the LTR using a lambda-specific forward and an LTR-specific reverse primer, scored using an LTR-specific probe. Primer sequences and protocol were used from Tervo et al. [65]. For data analyses, genomic DNA from a SC-1 standard cell line, harboring three HIV-1-based vector integrations [66], was diluted over a range of concentrations covering three logs. Slope and y-intercept of the standard curve were used to determine proviral copies. PTBP2 served as a housekeeping control. To exclude plasmid contamination of vector supernatants, 5 µM Nevirapine was used as an RT inhibitor, for every time point with equivalent volumes of vector supernatants, and signals were subtracted from the total signal. For each sample, PCR controls, omitting the B1 forward and reverse primers or the Lambda-LTR forward primer, were performed in parallel during the first PCR stage. The approach omitting the Lambda-LTR forward primer did not reveal any signal. The signal achieved by omission of B1 forward and reverse primers was subtracted from the total signal for each sample.

### Western blot analysis

Cells were harvested, washed with PBS and lysed by using radioimmunoprecipitation assay buffer supplemented with proteinase inhibitors (Complete Mini, Roche, Mannheim, Germany). Samples (15 µg) were separated by sodium dodecyl sulfate-polyacrylamide gel electrophoresis (12.5%) and blotted onto nitrocellulose membranes (GE Healthcare Europe GmbH). Antibody probing was conducted with rabbit monoclonal anti-CypA (Cell Signaling Technology, Danvers, MA, USA) and rabbit polyclonal Erk2 (Santa Cruz Biotechnology) according to the manufacturer’s instructions. Goat anti-rabbit IgG conjugated with horseradish peroxidase was used as a secondary antibody. Quantitative detection was carried out using the Fusion Fx system (Peqlab GmbH/VWR Life Science Competence Center, Erlangen, Germany).

### Pre-transduction/abrogation experiments

iPSC were pre-transduced with LV.DsRed or GV.DsRed at an MOI of 1000. After spin-inoculation cells were incubated at 37 °C and 5% CO_2_. After 6 h incubation time, iPSC were washed twice with PBS and a second transduction with LV was performed. LV encoding EGFP at an MOI of 100 were used for the second transduction. Flow cytometry analyses were conducted 4 days after transduction experiments.

### Statistical analysis

Data were expressed as mean ± standard deviation. Where appropriate, we used one-way ANOVA with Tukey-Kramer post hoc test to adjust for multiplicity effects. When comparing all groups to one control group, we utilized one-way ANOVA with Dunnett post hoc test. For time course analyses, we utilized repeated measures one-way ANOVA with appropriate post hoc test. The unpaired *t* test was performed for comparison of two groups. In case of significantly different variances between the groups, the unpaired *t* test with Welch’s correction was applied. p values of ≤ 0.05 were considered significant (*), ≤  0.01 very significant (**), ≤  0.001 extremely significant (***), and ns was considered not significant. Supplementary material and methods are described in Additional file 6.

## Additional files

**Additional file 1.** MG132 exhibits cytotoxicity at doses ≥ 0.2 µM in iPSC. iPSC were treated with MG132 at indicated concentrations (n = 3). **(A)** Cell counts are shown after 12 hours. **(B)** Cells were stained for AnnexinV and PI after 12 hours and percentages of fluorescence positive cells are shown. Camptothecin served as a positive control for cytotoxicity.

**Additional file 2.** CSA exhibits no changes in cell growth or apoptosis at a concentration of 10 µM in iPSC. iPSC were treated with CSA at indicated concentrations and washed after 12 hours (n = 3). **(A)** Cell counts are shown after 48 hours. **(B)** Cells were stained for AnnexinV and PI after 48 hours and percentages of fluorescence positive cells are shown. Camptothecin served as a positive control for cytotoxicity.

**Additional file 3.** iPSC transduced with wt or N74D capsid mutants exhibit comparable late RT levels. iPSC were transduced with LV N74D capsid mutant and wt at an MOI of 100. Late RT products were analyzed with TaqMan-based quantitative real-time PCR with 2^−∆∆Ct^ method 24 hours after transduction. Data are shown from 3 independent retroviral supernatants (n = 3) and as a ratio of late RT product level and plasmid contamination control, for which Nevirapine (Nev) was used, relative to endogenous PTBP2 level. The unpaired t-test was used for statistical analysis. ns p = 0.669.

**Additional file 4.** LV nuclear entry is impaired in iPSC. LV were applied to iPSC and CF-1 Mefs at an MOI of 100 in the presence of 10 µM CSA and/or 50 µM Raltegravir or an equal volume of DMSO as solvent control. Data are shown from three independent retroviral supernatants (n = 3). **(A)** Relative 2-LTR circle levels were determined 48 hours after transduction and analyzed with TaqMan-based quantitative real-time PCR with the 2^−∆∆Ct^ method, and normalized to endogenous PTBP2 copies. Data are shown relative to Mefs treated with DMSO. One-way ANOVA with Tukey-Kramer post-hoc test was used for statistical analyses. ns p = 0.8338; ** p = 0.0013; *** p ≤ 0.001. **(B)** Relative vector copies were determined 21 days after transduction and analyzed with TaqMan-based quantitative real-time PCR with the 2^−∆∆Ct^ method, and normalized to endogenous PTBP2 copies. Data are shown relative to Mefs treated with DMSO. One-way ANOVA with Tukey-Kramer post-hoc test was used for statistical analysis. *** p ≤ 0.001.

**Additional file 5.** Microarray analysis comparison of iPSC and fibroblasts reveals similar or even higher expression of a set of HIV-1 host co-factors and nucleoporins. Heat map is shown for 2 independent preparations of primary adult fibroblasts (Ad fib I + II), which served as parental fibroblasts for reprogramming, and different murine iPSC clones (#3, #2, #2EX). **(A)** Log_2_-intensity values for important HIV-1 host co-factors for nuclear entry and integration. **(B)** Log_2_-intensity values for a set of murine nucleoporins.

**Additional file 6.** Supplementary material and methods.
