# Correction to: Potent and reversible lentiviral vector restriction in murine induced pluripotent stem cells

**DOI:** 10.1186/s12977-017-0372-3

**Published:** 2017-10-18

**Authors:** Franziska K. Geis, Melanie Galla, Dirk Hoffmann, Johannes Kuehle, Daniela Zychlinski, Tobias Maetzig, Juliane W. Schott, Adrian Schwarzer, Christine Goffinet, Stephen P. Goff, Axel Schambach

**Affiliations:** 1 Institute of Experimental Hematology, Hannover Medical School, Carl-Neuberg-Str. 1, Hannover, Germany; 2 REBIRTH Cluster of Excellence, Hannover Medical School, Hannover, Germany; 3 Institute of Experimental Virology, TWINCORE, Centre for Experimental and Clinical Infections Research, Hannover, Germany; 4 Department of Biochemistry and Molecular Biophysics, Columbia University Medical Center, New York, NY USA; 5 Department of Microbiology and Immunology, Columbia University Medical Center, New York, NY USA; 6 Howard Hughes Medical Institute, Columbia University Medical Center, New York, NY USA; 7 Division of Hematology/Oncology, Children’s Hospital Boston, Harvard Medical School, Boston, MA USA

## Correction to: Retrovirology (2017) 14:34 DOI 10.1186/s12977-017-0358-1

The authors wish to apologize for an error within the scale bar of the microarray heatmap in Additional File 5 of the supplementary information. Two values were incorrectly displayed on the scale bar (11 instead of 10 and 13 instead of 12). The scale bar has been changed to reflect these corrections, and the numeric values are now evenly spaced.

This correction has no influence on any analyses or interpretation of the published data and does not affect the validity of the results or conclusions of this paper.

## Additional file

**Additional file 5.** Microarray analysis comparison of iPSC and fibroblasts reveals similar or even higher expression of a set of HIV-1 host co-factors and nucleoporins. Heat map is shown for 2 independent preparations of primary adult fibroblasts (Ad fib I + II), which served as parental fibroblasts for reprogramming, and different murine iPSC clones (#3, #2, #2EX). **(A)** Log_2_-intensity values for important HIV-1 host co-factors for nuclear entry and integration. **(B)** Log_2_-intensity values for a set of murine nucleoporins.
