# Supplementary material for: Potent and reversible lentiviral vector restriction in murine induced pluripotent stem cells
Source: Retrovirology. 2017 May 31;14:34. doi: 10.1186/s12977-017-0358-1 (PMC5452410; doi:10.1186/s12977-017-0358-1)
Supplement: Supplementary file 6 — Additional file 6. Supplementary material and methods. [file 12977_2017_358_MOESM6_ESM.docx]

**Additional file 6: Supplementary material and methods**

**Additional file 1: Cytotoxicity assay to test MG132 effects.** iPSC were separated with 0.5% Trypsin-EDTA in PBS and 2x10^5^ cells were treated with MG132 at indicated concentrations. Cells were counted 12 hours after drug application. 1-2x10^5^ cells were washed with 4°C PBS twice and resuspended in 100 µl AnnexinV binding buffer (BD Biosciences, Heidelberg, Germany) and stained with FITC-labeled AnnexinV antibody (BioLegend, San Diego, CA, USA) at a concentration of 5 µg/test for 20 minutes at room temperature in the dark. After staining, samples were diluted with an appropriate volume of AnnexinV binding buffer for flow cytometry measurement, and PI (1µg/mL) was added shortly before measurement.

**Additional file 2: Cytotoxicity assay to CSA effects.** iPSC were separated with 0.5% Trypsin-EDTA in PBS and 2x10^5^ cells were treated with CSA at indicated concentrations and were washed after 12 hours treatment. Cells were counted 48 hours after drug application. 1-2x10^5^ cells were washed with 4°C PBS twice and resuspended in 100 µl AnnexinV binding buffer (BD Biosciences) and stained with FITC-labeled AnnexinV antibody (BioLegend) at a concentration of 5 µg/test for 20 minutes at room temperature in the dark. After staining, samples were diluted with an appropriate volume of AnnexinV binding buffer for flow cytometry measurement, and PI (1µg/mL) was added shortly before measurement.

**Additional file 3: 2-LTR and integration analyses of iPSC and Mefs.** LV were applied to iPSC and CF-1 Mefs at an MOI of 100 and treated with 10 μM CSA and/or 50 μM Raltegravir or an equal volume of DMSO as solvent control. DNA was isolated 48 hours after transduction and 2-LTR circles were determined with TaqMan-based quantitative real-time PCR with the 2^–ΔΔCt^ method, and normalized to endogenous PTBP2 copies. Mean vector copy numbers were determined by normalization of the EGFP signal (primer sequences: for: 5’GACGTAAACGGCCACAAGTT3’, rev: 5’AAGTCGTGCTGCTTCATGTG3’, probe: 5’GCAAGCTGACCCTGAAGTTC3’) to endogenous PTBP2 DNA copy numbers and quantification was performed 21 days after transduction to avoid measuring non-integrated RT products. Determination of mean vector copy numbers per cell was based on a reference plasmid containing EGFP and PTBP2 sequences. The Fast Advanced Master Mix 2x (Applied Biosystems) was used. PCR for 2-LTR circles and mean vector copy numbers was performed at 50°C for 2 min and at 95°C for 20 sec followed by 60 cycles of 5 sec at 95°C, 20 sec at 56°C and 20 sec at 65°C.

**Additional file 4: Late RT level determination of N74D mutant and wt LV.** iPSC were transduced with LV N74D capsid mutant and wt LV at an MOI of 100. Nevirapine (5 µM) was added during and after transduction to inhibit RT and served as a plasmid contamination control. Late RT products were analyzed with TaqMan-based quantitative real-time PCR with the 2^–ΔΔCt^ method 24 hours after vector application. Data were normalized to endogenous PTBP2 copy numbers.

**Additional file 5: Microarray analyses of murine iPSC and parental fibroblasts.** iPSC clones #2, #2EX (excised reprogramming cassette) and #3 (C57BL/6 p14f/f, wt version without conditional knockout of the p14 gene) as well as two different adult fibroblast preparations of C57BL/6 p14f/f were utilized to perform microarray analysis. RNA quality was monitored using the Agilent 2100 Bioanalyzer. RNA amplification was accomplished with by the Applause WT-Amp ST System (NuGEN Technologies, Bemmel, The Netherlands). Amplified cDNA was fragmented and biotin labeled using the Encore Biotin Module (NuGEN Technologies) according to the manufacturer’s protocol. Fragmented and labeled cDNA was hybridized to GeneChip Mouse Gene 1.0 ST Array (Affymetrix, High Wycombe, UK). Microarray data are available at the GEO database (GSE97152). Bioinformatic analysis was performed using R 3.3.1 and Bioconductor 3.4. Raw data was normalized using robust multichip average (RMA) [1]. Differential expression analysis was performed using linear models for microarrays in the Bioconductor package LIMMA [2].

1. Gautier L, Cope L, Bolstad BM, Irizarry RA: **Affy - Analysis of Affymetrix GeneChip data at the probe level**. *Bioinformatics* 2004, **20**:307–315.

2. Ritchie ME, Phipson B, Wu D, Hu Y, Law CW, Shi W, Smyth GK: **limma powers differential expression analyses for RNA-sequencing and microarray studies**. *Nucleic Acids Res* 2015, **43**:e47.
