# Supplementary figures and images for: Correction to: Potent and reversible lentiviral vector restriction in murine induced pluripotent stem cells
Source: Retrovirology. 2017 Oct 18;14:48. doi: 10.1186/s12977-017-0372-3 (PMC5648417; doi:10.1186/s12977-017-0372-3)

**A**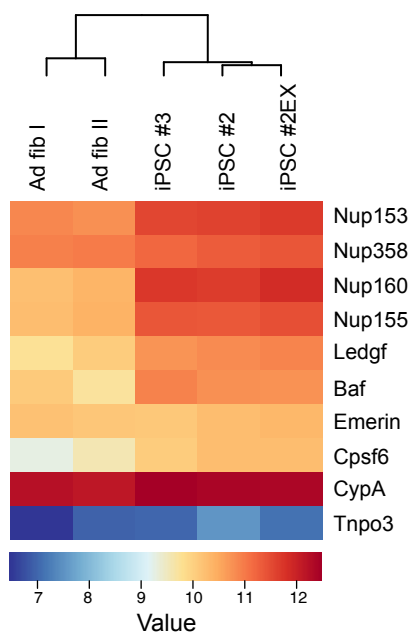**B**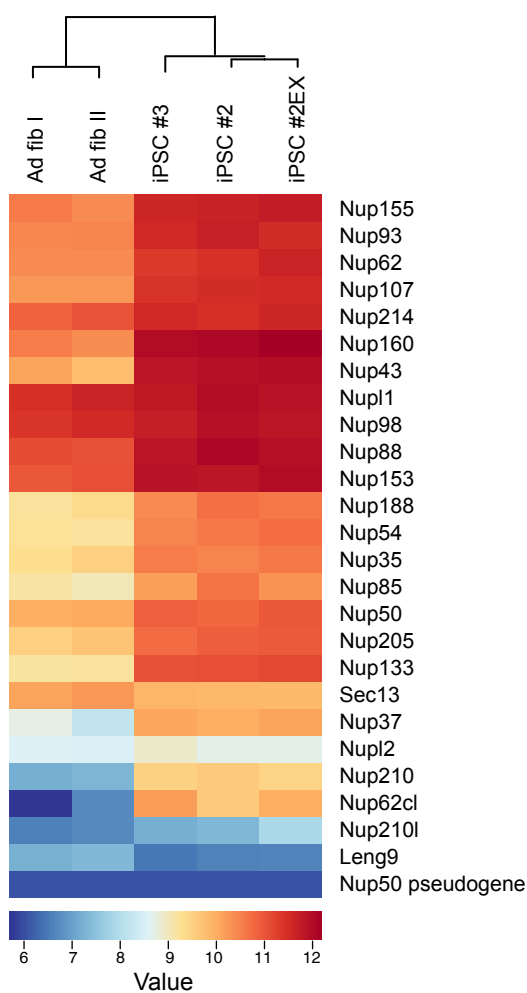

Supplement: Supplementary file 1 — Additional file 5. Microarray analysis comparison of iPSC and fibroblasts reveals similar or even higher expression of a set of HIV-1 host co-factors and nucleoporins. Heat map is shown for 2 independent preparations of primary adult fibroblasts (Ad fib I + II), which served as parental fibroblasts for reprogramming, and different murine iPSC clones (#3, #2, #2EX). (A) Log2-intensity values for important HIV-1 host co-factors for nuclear entry and integration. (B) Log2-intensity values for a set of murine nucleoporins. [file 12977_2017_372_MOESM1_ESM.pdf]
